# Saporin-conjugated tetramers identify efficacious anti-HIV CD8+ T-cell specificities

**DOI:** 10.1371/journal.pone.0184496

**Published:** 2017-10-11

**Authors:** Ellen M. Leitman, Christine D. Palmer, Søren Buus, Fabian Chen, Lynn Riddell, Stuart Sims, Paul Klenerman, Asier Sáez-Cirión, Bruce D. Walker, Paul R. Hess, Marcus Altfeld, Philippa C. Matthews, Philip J. R. Goulder

**Affiliations:** 1 Department of Paediatrics, University of Oxford, Oxford, United Kingdom; 2 Harvard Medical School, Boston, Massachusetts, United States of America; 3 Ragon Institute of MGH, MIT and Harvard, Cambridge, Massachusetts, United States of America; 4 Laboratory of Experimental Immunology, Faculty of Health Sciences, University of Copenhagen, Copenhagen, Denmark; 5 Department of Sexual Health, Royal Berkshire Hospital, Reading, United Kingdom; 6 Integrated Sexual Health Services, Northamptonshire Healthcare NHS Trust, Northampton, United Kingdom; 7 Institute of Virology, University of Zurich, Zurich, Switzerland; 8 NIHR Biomedical Research Centre, John Radcliffe Hospital, University of Oxford, Oxford, United Kingdom; 9 Nuffield Department of Medicine, University of Oxford, Oxford, United Kingdom; 10 Institut Pasteur, Unité HIV, Inflammation et Persistance, Paris, France; 11 HIV Pathogenesis Programme, The Doris Duke Medical Research Institute, University of KwaZulu-Natal, Durban, South Africa; 12 Immunology Program, Department of Clinical Sciences, North Carolina State University College of Veterinary Medicine, Raleigh, North Carolina, United States of America; 13 Virus Immunology Unit, Heinrich-Pette-Institut, Hamburg, Germany; 14 Department of Infectious Diseases and Microbiology, Oxford University Hospitals NHS Foundation Trust, John Radcliffe Hospital, Oxford, United Kingdom; National Institute of Allergy and Infectious Diseases, UNITED STATES

## Abstract

Antigen-specific T-cells are highly variable, spanning potent antiviral efficacy and damaging auto-reactivity. In virus infections, identifying the most efficacious responses is critical to vaccine design. However, current methods depend on indirect measures or on *ex vivo* expanded CTL clones. We here describe a novel application of cytotoxic saporin-conjugated tetramers to kill antigen-specific T-cells without significant off-target effects. The relative efficacy of distinct antiviral CD8+ T-cell specificity can be directly assessed via antigen-specific CD8+ T-cell depletion. The utility of these reagents is demonstrated here in identifying the CD8+ T-cell specificity most effective in preventing HIV progression in HIV-infected HLA-B*27-positive immune controllers.

## Introduction

The notion of selective T-cell depletion, most frequently aiming to purge autoreactivity, has recently gained substantial traction in the immunological field [1–8]. The development of fluorescently-labeled tetrameric peptide-MHC complexes (tetramers) allowed binding and visualisation of antigen-specific T-cells [9–11] and has led to the generation of modified tetramers that are coupled to a toxin, such as a ribosome inactivating protein saporin (SAP), that can selectively kill antigen-specific cells of interest. Being highly specific for their cognate T-cells and rapidly internalised upon engagement of the TCR, peptide-MHC tetramers can deliver any coupled moiety in a very selective manner [12]. The potential to cause death of selected target cells makes SAP-conjugated tetramers (tet-SAP) a powerful tool not only to eliminate auto-reactive T-cells causing disease but also by which to identify antiviral T-cell specificities that are effective in preventing disease [4].

An elegant proof-of-concept study in the mouse-LCMV model exploited the idea of SAP-conjugated tetramers and demonstrated tetramer-mediated selective depletion of certain CD8+ T-cells *in vitro* and *in vivo* [4]. These cytotoxic tetramers were later used *in vivo* in further murine studies to delete diabetogenic T-cells [6], encephalopathogenic T-cells [5], minor histocompatibility HY-specific T-cells to prevent organ rejection [7], or to study ‘memory inflation’ [13].

To date, however, the tet-SAP technology has not been applied in human studies. We here set out to demonstrate that this tool can be used to selectively deplete HIV-specific CD8+ T-cells *in vitro*, and thereby to evaluate the contribution and efficacy of a particular CD8+ T-cell specificity to viral inhibition. Although the importance of CD8+ T-cells in HIV control has been established for some decades [14, 15], a long-standing question–and one that is critical to HIV vaccine design–remains, namely, which of the many different HIV-specific responses are effective in mediating effective and durable immune control of HIV infection.

## Materials and methods

### Study subjects

Specimens from adult subjects from the following cohorts were used in our study:

1. Thames Valley cohort [16]: chronically HIV-infected ART-naïve adults were recruited from the Royal Berkshire Hospital, Reading, UK; Northampton General Hospital, Northampton, UK; Churchill Hospital, Oxford, UK; and Wycombe Hospital, High Wycombe, UK. This study was approved by the Institutional Review Board of the University of Oxford and all subjects provided written informed consent for participation in the study.
2. Acute HIV Infection cohort [17, 18]: acutely HIV-infected adults were recruited at the Massachusetts General Hospital, Brigham and Women’s Hospital and the Fenway Institute of the Beth Israel Deaconess Medical Center, Boston, MA, USA. Primary infection was classified using Fiebig staging, as previously described [19]. Subject AC198 from this cohort was studied. He is an adult Caucasian male, enrolled in 2005 during early acute infection (estimated 10–15 days post-infection, Fiebig II [19]) and received six months of ART that was ceased as part of randomly assigned treatment interruption. This study was approved by the Massachusetts General Hospital Institutional Review Board and all subjects provided written informed consent for participation in the study.
3. The Study of the Consequences of Protease Inhibitor Era (SCOPE) cohort [20]: chronically HIV-infected ART-naïve adults recruited at the University of California, San Francisco, USA. This study was approved by the Institutional Review Board of the University of California, San Francisco and all subjects gave written informed consent.

HIV plasma viral load was measured by the Roche Amplicor version 1.5 assay with with COBAS AmpliPrep or TaqMan 48 for the Acute HIV Infection cohort or with COBAS Amplicor for the other cohorts. CD4+ T-cell counts were determined by flow cytometry using standard clinical protocol. CD4+ T-cell counts and viral loads were measured on clinical grounds and the data were supplied by the Centre of Recruitment.HLA typing was performed using a locus specific PCR amplification strategy and a heterozygous DNA sequencing methodology for exon 2 and 3 of the class I PCR amplicon [21].

### Tetramer generation

Peptide-MHC tetramers conjugated to fluorophores were generated as previously described, using streptavidin-PE or APC to tetramerise biotinylated peptide-MHC class I monomers [11]. Efficiency of tetramerisation was confirmed by staining with anti-mouse Ig κ beads (BD Biosciences) with an anti-HLA antibody, followed by tetramer staining. Cytotoxic saporin-conjugated tetramers were produced by the same method using streptavidin-saporin (Advanced Targeting Systems) to tetramerise peptide-MHC monomers according to the published approach [4]. Briefly, these modified tetramers are coupled to a toxin, ribosome-inactivating protein saporin (SAP), that can selectively kill antigen-specific cells of interest and thereby evaluate the contribution of a particular CD8+ T-cell specificity to viral inhibition [4–7, 13]. Biotinylated peptide-MHC class I monomers generated as per standard published approach [11] were tetramerised by stepwise addition (1/10^th^ volume every 10 minutes) of streptavidin-SAP (Advanced Targeting Systems) at a 1:1 molar ratio of biotinylated MHC to biotin binding sites [22]. Between additions, tetramerisation reaction was left on a rotor at 4°C protected from light.

### Tetramer staining

For staining with fluorescently-conjugated tetramers, 0.5-1x10^6^ cells per stain were washed with PBS, incubated with relevant tetramers for 30 minutes at room temperature, washed, further incubated with fluorochrome-conjugated antibodies for 15–20 minutes at room temperature in the dark and fixed in 2% formaldehyde solution at 4°C. Controls included cells incubated with no tetramer and with HLA-mismatched tetramers; these were used to set up negative gates during analysis. For staining with SAP-conjugated tetramers, cells were incubated with tetramers for 30 minutes at room temperature, washed, fixed and permeabilised with BD Cytofix/Cytoperm kit (BD Biosciences) and then incubated with antibodies, including a secondary anti-SAP antibody (Advanced Targeting Systems). Controls included cells incubated with no tetramer, HLA-mismatched SAP-conjugated tetramers and free unconjugated SAP. Negative gates during analysis were set up based on no tetramer and HLA-mismatched tetramer stainings. Samples were analysed in FlowJo version 9.7.6 (Tree Star, Inc.) and hierarchically gated on singlets, lymphocytes, live cells and CD3+ cells and gated on tetramer-specific CD8+ cell populations. Antibodies used in these experiments: αSAP-Alexa488 (Advanced Targeting Systems); αCD3-Pacific Orange (Invitrogen); αCD3-Brilliant Violet 421, αCD4 OKT4-APC, αCD4 OKT4-FITC and αCD8-PE/Cy7 (BioLegend); αCD8-V450 (BD Biosciences); αHLA-APC (BD Biosciences); and LIVE-DEAD fixable near-IR marker (Life Technologies).

### Tetramer internalisation experiments

To establish the kinetics of tetramer interactions with human PBMCs, internalisation experiments were performed as previously described [4]. Briefly, cells were incubated with tetramers at 4°C for 30 minutes to allow binding, further incubated at either 37°C or 4°C to promote or inhibit internalisation, respectively, for up to 3 hours, stripped of external fluorescence with 0.5M NaCl/0.5M acetic acid (pH 2.5) and immediately acquired by flow cytometry.

### Timecourse of cell-depletion mediated by tetramer-SAP

To establish the timecourse and efficiency of selective tetramer-mediated cell depletion, PBMCs or expanded CD8+ T-cells were treated with tetramer-SAP (5–10 nM) for 2 hours at 37°C in R10, washed 3 times with R10 and further cultured in R10 (PBMCs) or R10/50 (expanded CD8+ T-cells) [4]. Control treatments included: free SAP, HLA-mismatched tetramer-SAP, tetramer-PE and no treatment. Every 24 hours aliquots of cells from each condition were stained with the relevant fluorochrome-conjugated tetramer and analysed by flow cytometry. For comparison with the conventional cell-depletion method using anti-PE magnetic beads [23], cells were incubated with tetramer-PE for 30 minutes on ice to allow surface binding and inhibit internalisation; tetramer-PE-bound cells were then removed using anti-PE beads (positive selection) (EasySep, StemCell Technologies) according to the manufacturer’s protocol.

### Viral inhibition assay

To evaluate the effect of tet-SAP-mediated depletion of epitope-specific CD8+ T-cells on their antiviral capacity, we modified previously described viral inhibition assay [24] as follows. We used HIV-permissive H9 cell line transfected with the HLA-B*27:05 gene (a generous gift from Otto Yang) as targets; controls included H9 cells expressing HLA-B*57:03 or HLA-untransfected. As effector cells, we treated PBMCs with a monoclonal CD3.4 antibody bi-specific for CD3 and CD4 (the NIH AIDS Reagent Program) to eliminate CD4+ T cells and expand CD8+ T-cells [25–27]. Expanding CD8+ T-cells were cultured for 10–20 days to achieve >90% purity and sufficient numbers. 24–48 hours prior to the inhibition assay, CD8+ T-cells were treated with tet-SAP or a control (untreated, HLA-mismatched tet-SAP, tet-PE, free SAP) as described above. When cell numbers allowed, cell-depletion was confirmed by tetramer staining. For the initial setup of the inhibition assay, target cells were infected with pre-titrated NL4-3-GFP [16] by spinoculation for 1 hour, incubated at 37°C for 1 hour, repeatedly washed and further cultured with or without effector cells at an appropriate effector to target cell ratio in duplicates or triplicates. Every 2–3 days, the cultures were fed and stained to assess live CD4+ GFP+ cells. % GFP+ uninfected target cells served as a background, subtracted from all values. HIV-suppressive capacity was calculated at the time of the peak of viral growth as follows [24]: suppressive capacity = log_10_(%GFP+ infected target cells without effector cells / %GFP+ target cells with effector cells). Analysis was performed in GraphPad Prism for Mac OSX, 5.0c (GraphPad Software).

### ELISPOT assay

Interferon-γ ELISPOT assays were performed as previously described [28, 29]. Peptides were generated by Schafer-N.

### Sanger sequencing of proviral genome

Genomic DNA was extracted from PBMCs using QIAmp DNA Mini kit following manufacturer’s protocol (Qiagen). Full length HIV genome was amplified in two overlapping fragments, purified and sequenced as previously described [30]. This was confirmed by sequencing of HIV protein-long fragments as previously described [31]. All sequencing was done using BigDye Terminator v3.1 Ready Reaction mix (Applied Biosystems) and analysed using Sequencher v4.8 (Gene Codes Corp.).

## Results

### Recognition and internalisation of conventional and cytotoxic tetramers

**Fig 1:**
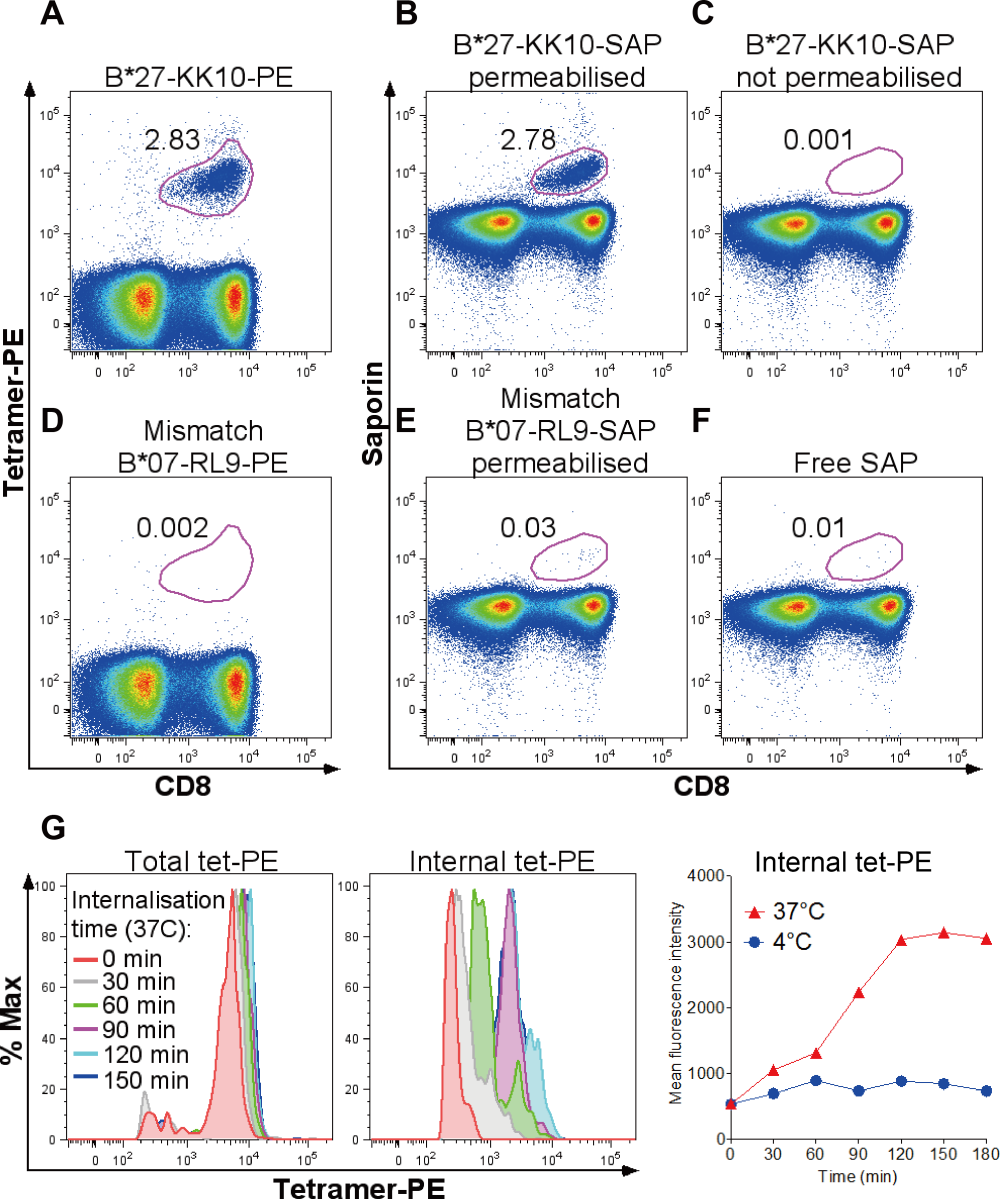
Recognition and internalisation of conventional and cytotoxic tetramers. **(A-F)** Representative dot plots of PBMC staining in an HIV-infected HLA-B*27:05-positive subject with conventional fluorescently labeled tetramer HLA-B*27:05-Gag-KK10-PE **(A)** or SAP-coupled HLA-B*27:05-Gag-KK10-SAP tetramer, detected with a secondary Alexa Fluor 488 anti-SAP antibody in permeabilised **(B)** or not permeabilised **(C)** cells. Absence of non-specific binding of HLA-mismatched tet-PE **(D)** and tet-SAP **(E)** and free unconjugated SAP **(F)** is shown. Gated on live CD3+ cells around CD8+tet+ cells; numbers indicate % tet+ cells (of CD8+). **(G)** Kinetics of tetramer binding and internalisation, determined by measurements of total and internal tetramer fluorescence in cells at internalisation-promoting (37°C) or internalisation-inhibiting (4°C) conditions. No further increase in internal fluorescence is observed after 90–120 minutes of internalisation time, suggesting that nearly all cognate metabolically active (37°C) CD8+ T-cells have internalised the tetramer. PBMC from an HIV-negative healthy donor with an EBV-HLA-A*02:01-GL9 response were used here. ‘Total tet-PE’ = surface-bound and internal tetramer fluorescence measured in cells not stripped of any surface-bound tetramer; ‘internal tet-PE’ = internal tetramer fluorescence measured in acid-stripped cells. A-F, representative of 8 independent experiments with PBMC from different donors. G, representative of 2 independent experiments with cells from two individuals.

We first confirmed that SAP conjugation to tetramers did not compromise tetramer recognition and internalisation by cognate CD8+ T-cells. Stainings with conventional, fluorescently labelled peptide-MHC tetramers (tet-PE or tet-APC) and with cytotoxic tetramers (tet-SAP) of the same specificity showed comparable levels of responses (S1 Fig). An example in Fig 1A and 1B illustrates that a similar level of the immunodominant Gag-KK10 (^263^KRWIILGLNK^272^) response was detected when PBMCs from an HLA-B*27:05-positive, HIV-infected donor were stained with either KK10-PE or KK10-SAP. Visualisation of tet-SAP-specific cells required permeabilisation and staining with an anti-SAP fluorescent antibody following incubation with tet-SAP (Fig 1B and 1C). HLA-mismatched tetramers (in this case HLA-B*07:02-specific tetramers) showed low background staining (≤0.03% of CD8+ T-cells), similar to conventional tetramers (Fig 1D and 1E). Free SAP, not conjugated to tetramers and therefore lacking the means of cell entry, did not show CD8+ T-cell binding or internalisation (Fig 1F). The kinetics of tetramer binding and internalisation was strongly temperature-dependent (Fig 1G), consistent with murine studies [4], and reached maximum within two hours of tetramer-PBMC incubation at 37°C.

### Tet-SAP effectively and specifically depletes cognate CD8+ T-cells

**Fig 2:**
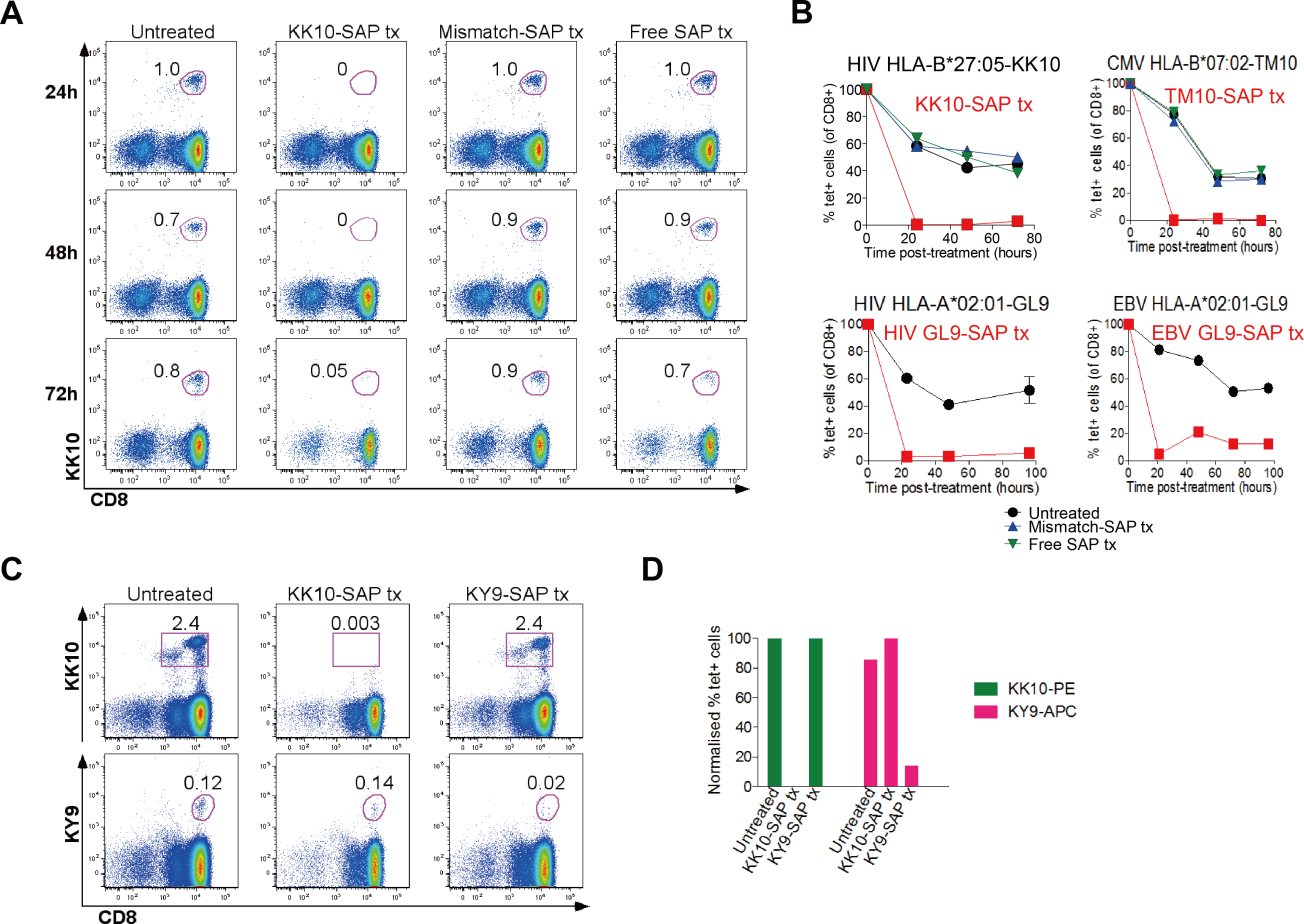
Tet-SAP effectively and specifically depletes cognate CD8+ T-cells. **(A)** Representative dot plots of a time course, showing depletion of HIV HLA-B*27:05-Gag-KK10-specific CD8+ T-cells mediated by KK10-SAP. At time -2 hours, cells were left untreated or treated with KK10-SAP, mismatch-SAP or free SAP for 2 hours at 37°C, washed and left in media (time = 0 hours). Every 24 hours aliquots of cells from each condition were stained with the relevant fluorochrome-conjugated tetramer and analysed by flow cytometry. Gated on live CD3+ cells around CD8+tet+ cells; numbers indicate % tet+ cells (of CD8+) assessed at indicated times after treatment. **(B)** Graphical representation of 4 depletion time courses (performed as in A) with tet-SAP of different HIV, CMV and EBV specificities and restricted by different HLA class I molecules using cells from different individuals with the corresponding specificities. At time -2 hours cells were treated with tetramer-SAP of interest (red lines and symbols): HIV HLA-B*27:05 KK10-SAP (top left), CMV HLA-B*07:02 TM10-SAP (top right), HIV HLA-A*02:01 GL9-SAP (bottom left), or EBV HLA-A*02:01 GL9-SAP (bottom right). Controls included: untreated cells (black lines and symbols), treated with mismatch-SAP tetramer (blue lines and symbols; when sufficient cell numbers were present), or treated with free SAP (green lines and symbols, when sufficient cell numbers were present). % tet+ cells is normalised to baseline pre-treatment levels (time = -2h). Tx = treatment. **(C,D)** KK10-SAP-mediated depletion (assessed 48 hours post-treatment) of HLA-B*27:05-restricted KK10-specific CD8+ T-cells does not have an off-target effect on HLA-B*27:05-restricted KY9-specific CD8+ T-cells, while KY9-SAP-mediated elimination of KY9-specific cells does not affect KK10-specific cells. A,B, representative of at least 10 separate experiments with cells from different individuals with different HLA types and with tetramers of different specificities. C,D, representative of 3 independent experiments with cells from different HIV-positive HLA-B*27:05-positive donors.

We next assessed whether cytotoxic tetramers could deplete specific populations of human CD8+ T-cells. An illustrative example in Fig 2A, involving the same HLA-B*27:05-KK10 tetramers, shows staining of PBMCs with conventional KK10-PE tetramers following either no treatment, or treatment with KK10-SAP tetramer, HLA-mismatched tetramer or free SAP. This demonstrated that, by 24 hours post-treatment, nearly all KK10-specific cells had been depleted by tet-SAP, and did not re-emerge later in the time course (up to 72hrs). Similar observations were made in relation to four other SAP-conjugated tetramers, to represent a range of different HLA class I types and to include CD8+ T-cell responses to CMV and EBV in addition to HIV (Fig 2B).

To address the possibility that tetramer-mediated depletion of CD8+ T-cells of one specificity has any off-target effects on other CD8+ T-cell specificities, in particular those restricted by the same HLA class I molecule, we studied the impact of the HLA-B*27:05-KK10 tet-SAP on CD8+ T-cells recognising the HLA-B*27:05-restricted, subdominant HIV Pol epitope KY9 (^901^KRKGGIGGY^909^) [16]. Treatment with KK10-SAP resulted in effective depletion of KK10-specific cells, but did not affect KY9-specific cells (Fig 2C and 2D). Similarly, KY9-SAP-mediated depletion of KY9-specific cells did not have an effect on KK10-specific cells. These data underscore the high degree of specificity maintained by the cytotoxic tetramers.

### Contribution of KK10 to immune control of HIV determined by tet-SAP

**Fig 3:**
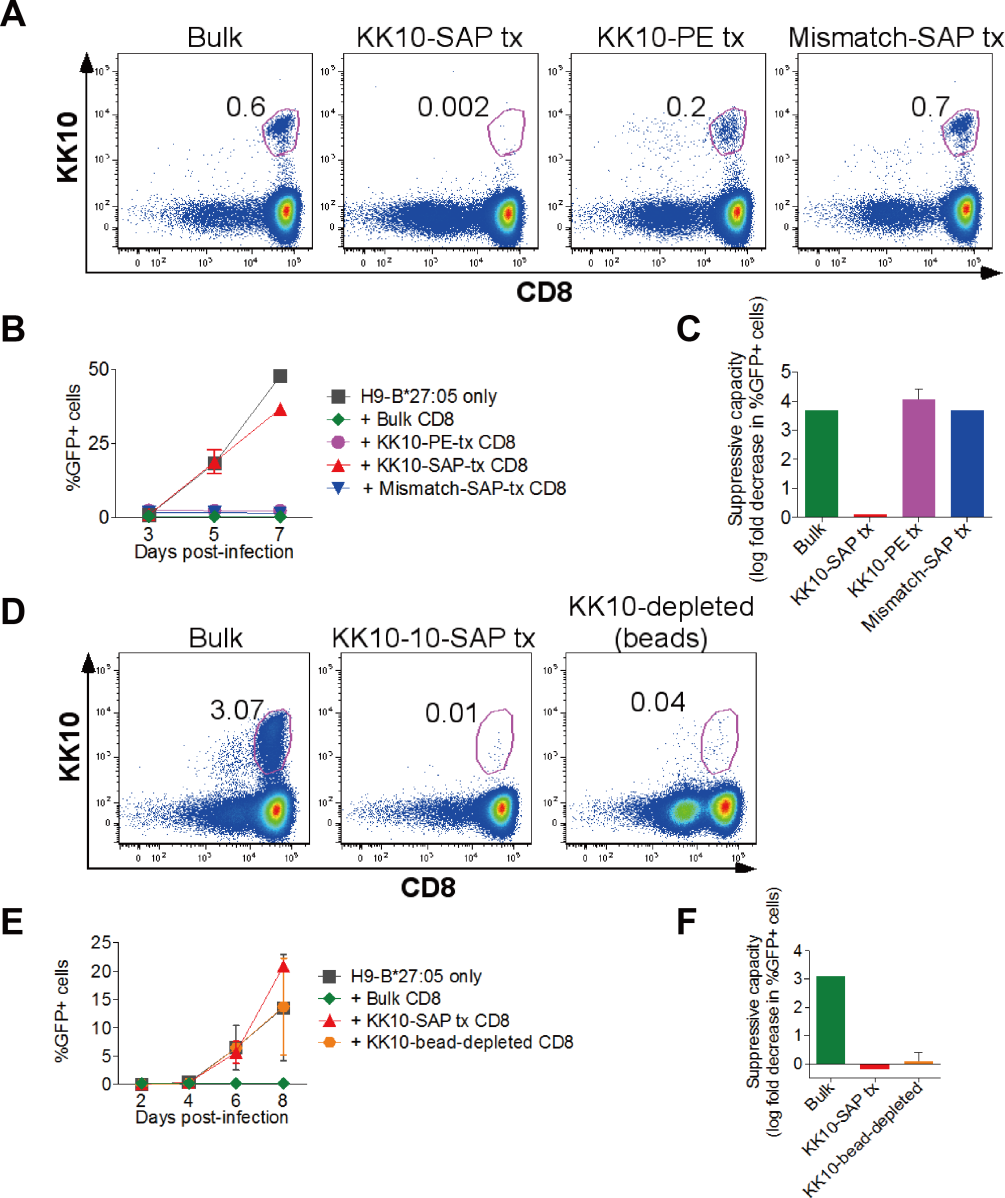
Contribution of KK10 to immune control of HIV determined by tet-SAP. Panels A-C and D-F show results with cells from two different HIV-infected HLA-B*27:05-positive donors. **(A)** Tetramer staining 48h post-treatment with KK10-SAP to confirm depletion of KK10-specific cells. Gated on live CD3+ cells around CD8+tet+ cells; numbers indicate % tet+ cells (of CD8+). **(B)** Viral replication in HLA-B*27:05-expressing H9 cells without or with added untreated bulk, KK10-SAP-treated, KK10-PE-treated or mismatch-tet-SAP-treated CD8+ T-cells. Infected cells were measured by NL4-3-GFP expression. **(C)** Suppressive capacity of bulk, KK10-SAP-treated or mismatch SAP-treated CD8+ T-cells. **(D)** Tetramer staining to confirm KK10-SAP-mediated depletion of KK10-specific CD8+ T-cells (48 hours post-treatment) or depletion of KK10-PE-stained cells with anti-PE magnetic beads. **(E)** Viral replication (as in B) in H9-HLA-B*27:05-positive infected target cells alone or with bulk, KK10-SAP-treated or KK10-bead-depleted CD8+ T-cells. **(F)** Suppressive capacity of bulk, KK10-SAP-treated or KK10-bead-depleted CD8+ T-cells. B,C,E,F, error bars represent s.e.m. Represents 2 separate experiments with two HLA-B*27:05-positive donors.

We next applied cytotoxic tetramers to determine the contribution of specific CD8+ T-cell responses to immune control of viral infections such as HIV. To evaluate the contribution of the HLA-B*27:05-KK10 response in an HIV-infected HLA-B*27:05-positive subject, we non-specifically expanded ‘bulk’ CD8+ T-cells from PBMCs using the bi-specific monoclonal CD3.4 antibody [25–27], and then treated them with either the KK10-tet-SAP or treatments that did not affect the HLA-B*27:05-KK10 CD8+ T-cells (no treatment, treatment with KK10-PE tetramer or with an HLA-mismatched tet-SAP; Fig 3A). We then assessed inhibition of HIV replication in HLA-B*27:05-expressing target cells by CD8+ T-cells with or without Gag-KK10 depletion. The example in Fig 3B and 3C illustrates that the CD8+ T-cells with unaffected HLA-B*27:05-KK10-specific cells inhibited viral replication by 3–4 log_10_ compared to the KK10-depleted CD8+ T-cells that lost the inhibitory capacity. Of note, treatment with KK10-PE tetramer resulted in a lower percentage of KK10-specific cells being detectable by tetramer staining 48 hours following the treatment, which was likely caused by the transient loss of their capacity to efficiently bind tetramers–a normal characteristic of CD8+ T-cell activation [32]. Importantly, however, KK10-PE treatment did not impact T-cell suppressive capacity, whereas KK10-SAP treatment substantially reduced viral inhibition (Fig 3B and 3C). These results strongly suggest that the KK10-specific cells had indeed been depleted by the cytotoxic KK10-SAP rather than become refractory to tetramer-PE binding and thus undetectable by tetramer staining. Additionally, as we demonstrated earlier, the potent toxic tetramer had been internalised (Fig 1) and no rebound KK10-specific T-cell population re-emerged for up to 72 hours post-treatment (Fig 2A and 2B).

In a separate experiment, we ran the same tet-SAP depletion procedure in parallel with depletion using anti-PE magnetic beads[23] (Fig 3D–3F). This showed that depletion efficiency and loss of viral inhibition were similar irrespective of the method of depletion (see Discussion below).

### Definition of antiviral efficacy of different CD8+ T-cell responses using tet-SAP

**Fig 4:**
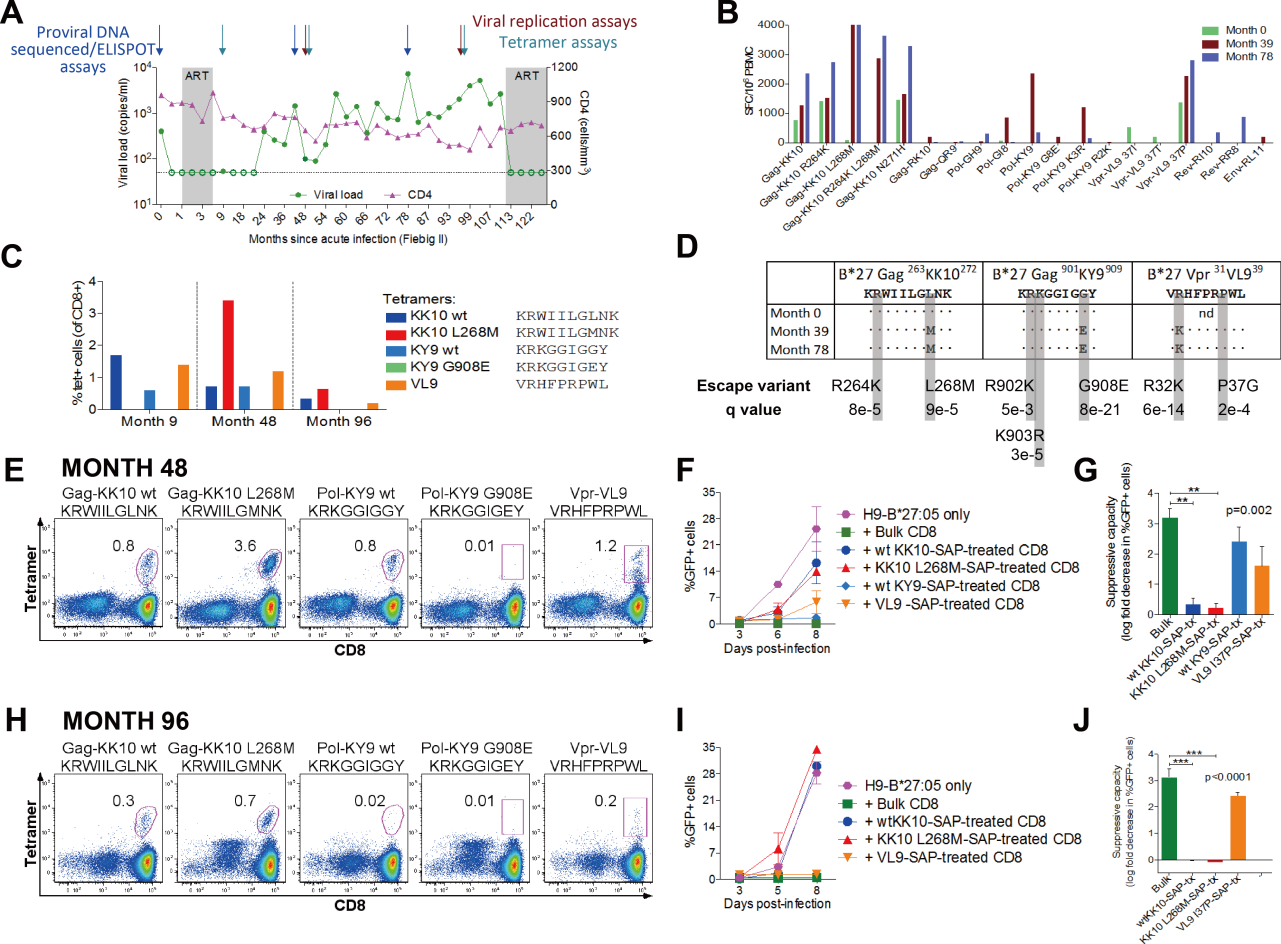
Definition of antiviral efficacy of different CD8+ T-cell responses using tet-SAP. All panels show data from one HIV-infected HLA-B*27:05-positive subject. **(A)** Viral loads, CD4+ T-cell counts with timepoints of assays indicated. **(B)** IFN-γ ELISPOT CD8+ T-cell responses to HLA-B*27-restricted optimal epitopes. Only responses >50 SFC/10^6^ PBMC are shown. **(C)** Frequency of antigen-specific cells of three dominant specificities determined by tetramer staining. **(D)** Proviral sequences of the three dominant responses. Known HLA-B*27:05-associated footprints and the significance of the associations (q value) are shown [33]. Nd, not done. **(E-G)** Results from timepoint 48 months post-infection; (H-J) results from timepoint 96 months post-infecition. **(E,H)** Dot plots showing tetramer staining of existing in this donor HLA-B*27:05-restricted responses at the timepoint assayed. Gated on live CD3+ cells around CD8+tet+ cells; numbers indicate % tet+ cells (of CD8+). **(F,I)** Viral replication in H9-HLA-B*27:05-positive infected target cells alone or with bulk CD8+ T-cells or CD8+ T-cells depleted of a particular specificity with tet-SAP. Infected cells were measured by NL4-3-GFP expression. **(G,H)** Suppressive capacity of bulk or tet-SAP-depleted CD8+ T-cells. F,G,I,J, error bars represent s.e.m. G,J, ANOVA with Dunnett’s Multiple comparisons test to compare suppressive capacity of bulk versus tet-SAP-treated CD8+ T-cells. *p<0.05, **p<0.01, ***p<0.001.

We next employed the tet-SAP reagents to compare simultaneously HIV-suppressive capacity of several CD8+ T-cell specificities following this approach (S2 Fig). We used samples from an HLA-B*27:05-positive HIV-infected donor who initially controlled viraemia and had three wild-type HLA-B*27-restricted dominant responses, as determined by IFN-γ ELISPOT assays and confirmed by tetramer staining (Fig 4A–4C): Gag-KK10, Pol-KY9 and Vpr-VL9 (^31^VRHFPRPWL^39^). Responses to the wild-type epitopes diminished over time, while viraemia progressively increased and well-characterised HLA-B*27-associated mutations emerged in the dominant responses (Fig 4D). Viral sequence encoding the Vpr epitope was not available for the earliest timepoint, but the R32K escape mutant present at months 39 and 78 is characteristically selected specifically in HLA-B*27-positive subjects [33].

We next evaluated the impact on viral inhibition of tet-SAP-mediated depletion of CD8+ T-cell responses present, studying two timepoints, 4 and 8 years after infection. At 4 years (48 months) post-infection, Pol-KY9 and Vpr-VL9 depletion made little difference to viral replication, while Gag-KK10 depletion significantly attenuated the ability of CD8+ T-cells to suppress viral replication, suggesting that the majority of bulk CD8+ T-cell-mediated inhibition was Gag-KK10-specific (Fig 4E–4G). At 8 years (96 months) post-infection (Fig 4H–4J), the contribution of the Pol-KY9 response to inhibition of viral replication was lost altogether; Vpr-VL9 continued not to contribute significantly to viral inhibition, and the Gag-KK10-specific cells contributed the most. Of note, the Gag-KK10 effect was seen with both the wild-type-specific and mutant L268M-specific tet-SAP mediated depletion (Fig 4G and 4J). This can be explained by cross-recognition of the variant by the wild-type-specific cells that are highly effective against both wild-type and L268M viruses, express an anti-apoptotic phenotype and are long-lived.[34, 35] Although in this case the subject initiated ART through choice after 9 years of infection, viral load was well contained for much of this time at <2,000 copies/ml, and absolute CD4 counts were maintained at high levels of 500–700 cells/mm^3^ until ART intervention. This is representative of 2 similar experiments involving HLA-B*27:05-positive HIV-infected individuals, showing that in subjects with low viral loads (e.g. 73 copies/ml, 518 copies/ml), inhibition of viral replication was highly dependent upon the Gag-KK10 response (S3 Fig).

## Discussion

In summary, we here show, for the first time, that cytotoxic saporin-conjugated tetramers can be used in *in vitro* studies with human cells. These tetramers bind and are internalised by cognate CD8+ T-cells, resulting in their effective elimination by as little as 24 hours. We did not observe an off-target effect and found that the tet-SAP approach is considerably simpler and less time-consuming than the conventional method using magnetic beads, especially if more than one CD8+ T-cell specificity is being assessed. These reagents can facilitate identification of effective HIV-specific CD8+ T-cell responses that could be induced by a successful vaccine, and can also be used in other viral infections such as CMV or HCV. Finally, as shown in murine studies [4], saporin-conjugated tetramers have the potential for *in vivo* depletions to be undertaken immunotherapeutically in humans.

## Supporting information

S1 Fig Similar levels of CD8+ T-cell responses are detected with both conventional tet-PE and tet-SAP. Spearman correlation of stainings of PBMC from 8 different donors with tetramers of different specificities and restricted by different HLA types.
(TIFF)

S2 Fig Workflow of the method to assess anti-HIV efficacy of different CD8+ T-cell specificities in human cells using tet-SAP. The proposed method consists of four main steps:
Identify CD8+ T-cell responses by IFN-γ ELISPOT and/or tetramer staining.Expand CD8+ T-cells with bi-specific CD3.4 monoclonal antibody and confirm targeted specificities by tetramer staining
2.1Include an anti-CD4 antibody in the panel to assess CD8+ T-cell purity.2.2Use this period to generate SAP-conjugated tetramers.2.3Prepare target cells: (i) if using HIV-permissive cell lines (e.g. H9, U937, T1), start the cultures a week before infection; (ii) if using primary CD4+ T cells, start their activation 3–4 days before superinfection.Remove desired specificities with tet-SAP and confirm by tetramer staining. Include controls (HLA-mismatched tet-SAP, free SAP).Perform viral inhibition assay using tet-SAP-treated CTL as effector cells. Use intracellular Gag-p24 staining or ELISA as a read-out if the virus used for infection does not have a GFP reporter.
(TIFF)

S3 Fig Examples of HIV-infected HLA-B*27:05-positive individuals with low viral loads in whom control of viral replication was dependant on Gg-KK10 response. Panels A,B show data for an HLA-B*27:05-positive controller with viral load of 73 copies/ml; panels C,D show data for another HLA-B*27:05-positive controller with viral load of 518 copies/ml. **(A,C)** Viral replication in H9-HLA-B*27:05-positive infected target cells alone or with bulk CD8+ T-cells or CD8+ T-cells depleted of Gag-KK10 specificity with tet-SAP. Infected cells were measured by NL4-3-GFP expression. **(B,D)** Suppressive capacity of bulk or KK10-tet-SAP-depleted CD8+ T-cells. Error bars represent s.e.m.
(TIFF)
