# Supplementary figures and images for: Saporin-conjugated tetramers identify efficacious anti-HIV CD8+ T-cell specificities
Source: PLoS One. 2017 Oct 11;12(10):e0184496. doi: 10.1371/journal.pone.0184496 (PMC5636067; doi:10.1371/journal.pone.0184496)

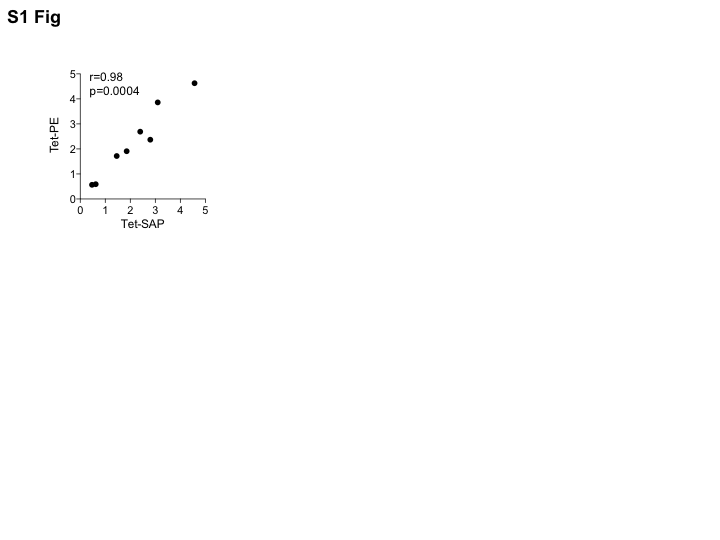

Supplement: S1 Fig — Spearman correlation of stainings of PBMC from 8 different donors with tetramers of different specificities and restricted by different HLA types. (TIFF) [file pone.0184496.s001.tiff]

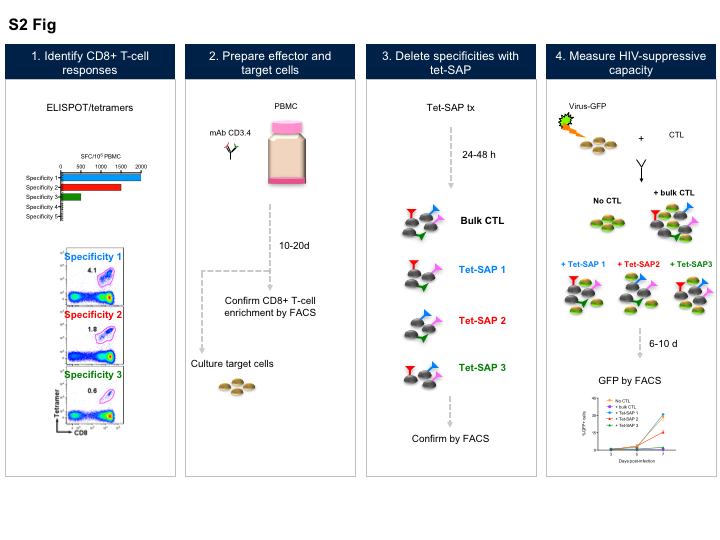

Supplement: S2 Fig — The proposed method consists of four main steps: Identify CD8+ T-cell responses by IFN-γ ELISPOT and/or tetramer staining.Expand CD8+ T-cells with bi-specific CD3.4 monoclonal antibody and confirm targeted specificities by tetramer staining 2.1Include an anti-CD4 antibody in the panel to assess CD8+ T-cell purity.2.2Use this period to generate SAP-conjugated tetramers.2.3Prepare target cells: (i) if using HIV-permissive cell lines (e.g. H9, U937, T1), start the cultures a week before infection; (ii) if using primary CD4+ T cells, start their activation 3–4 days before superinfection.Remove desired specificities with tet-SAP and confirm by tetramer staining. Include controls (HLA-mismatched tet-SAP, free SAP).Perform viral inhibition assay using tet-SAP-treated CTL as effector cells. Use intracellular Gag-p24 staining or ELISA as a read-out if the virus used for infection does not have a GFP reporter. (TIFF) [file pone.0184496.s002.tiff]

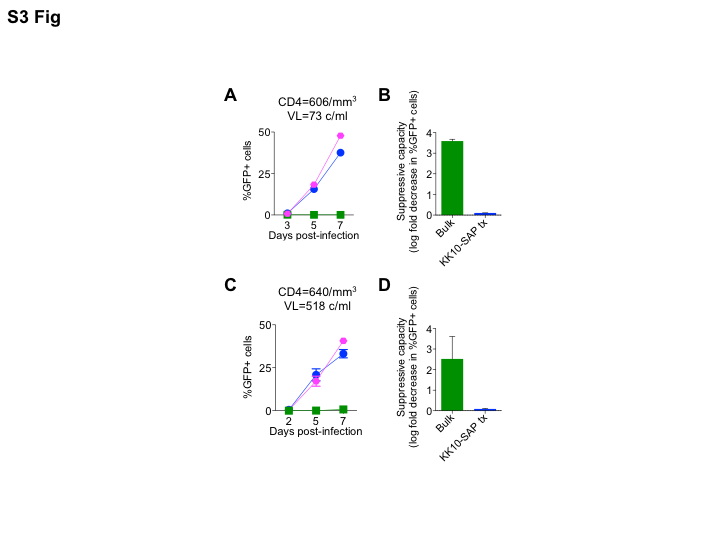

Supplement: S3 Fig — Panels A,B show data for an HLA-B*27:05-positive controller with viral load of 73 copies/ml; panels C,D show data for another HLA-B*27:05-positive controller with viral load of 518 copies/ml. (A,C) Viral replication in H9-HLA-B*27:05-positive infected target cells alone or with bulk CD8+ T-cells or CD8+ T-cells depleted of Gag-KK10 specificity with tet-SAP. Infected cells were measured by NL4-3-GFP expression. (B,D) Suppressive capacity of bulk or KK10-tet-SAP-depleted CD8+ T-cells. Error bars represent s.e.m. (TIFF) [file pone.0184496.s003.tiff]
